# Life Course Stressors and Cognitive Function: A Latent Profile Analysis Approach

**DOI:** 10.1093/geroni/igaf122.2565

**Published:** 2025-12-31

**Authors:** Jean Choi, Elizabeth Muñoz

**Affiliations:** The University of Texas at Austin, Texas, United States; University of Texas at Austin, Austin, Texas, United States

## Abstract

Psychosocial stressors are risk factors for poor cognitive health, but existing research has largely focused on testing associations between single stressor domains or on specific life stages, overlooking the combined role of stressors across the life course. Moreover, marginalized racial/ethnic groups may be more susceptible to the effects of some exposures compared to their counterparts. We aimed to identify life course stressor profiles and examine their associations with cognitive function using the Midlife in the United States study (n = 3,684; age: 32–84). Latent profile analysis identified three profiles of life course stressors based on 11 domains: High Everyday Discrimination, High Work Stress, and High Multi-Domain Stress. Generalized estimating equations tested associations between profile membership and cognitive function. Compared to the High Everyday Discrimination profile, the High Work Stress profile had better executive function (b = 0.07, SE = 0.04, p = .016). Among non-Hispanic Blacks, the positive association between High Work Stress and executive function was weaker than for non-Hispanic Whites (b = -0.41, SE = 0.17, p = .01). The High Multi-Domain Stress profile had higher executive function among Hispanics (b = 0.64, SE = 0.26, p = .013) and greater episodic memory for both non-Hispanic Black (b = 0.44, SE = 0.21, p = .01) and Hispanics (b = 0.61, SE = 0.30, p = .04) compared to non-Hispanic Whites. These results suggest that cognitive health outcomes may be differentially predicted by distinct patterns of life course stressors, and its associations may vary by race/ethnicity.

